# Predicting marital adjustment in cancer patients: The impact of attachment styles, disease type, and stage on marital duration

**DOI:** 10.1192/j.eurpsy.2025.1784

**Published:** 2025-08-26

**Authors:** M. Firoozi, M. Sharifi, C. A. Lewis

**Affiliations:** 1 Psychology, University of Tehran, Tehran; 2 Psychology, Islamic Azad University, Bandar Gaz Branch, Bandar Gaz, Iran, Islamic Republic Of; 3 Psychology, Leeds Trinity University, Leeds, United Kingdom

## Abstract

**Introduction:**

This study examined the interplay between marital relationships and individual adjustment to cancer, considering the influence of attachment styles and marital duration under high-stress conditions.

**Objectives:**

This research aims to elucidate how these factors modulate the marital quality and the psychological well-being of individuals and their partners.

**Methods:**

A total of 312 participants, comprising 223 cancer patients and 89 partners, completed the Golombok-Rust Inventory of Marital State and the Adult Attachment Inventory. Linear mixed-effects models were used to analyze the relationships between marital adjustment, attachment styles, and marital duration.

**Results:**

The findings indicated that females diagnosed with cancer reported significantly poorer marital adjustment compared to their male counterparts (both p < .05). Notably, secure attachment was significantly positively associated with better marital adjustment (β = .18, p = .016), while ambivalent attachment was significantly negatively associated with worse marital adjustment (β = -.11, p = .029). These patterns persisted across both patients and their partners, with no significant differences based on sex. Additionally, the impact of attachment styles on marital adjustment did not vary significantly with marital duration (all p > .05).

**Conclusions:**

These results underscore the complexity of the marital dynamics among couples confronting cancer. The data reveal that secure attachment enhances marital stability, whereas ambivalent attachment detracts from it, irrespective of the sex of the respondent or marital duration. This study adds to the body of literature by highlighting the nuanced ways in which attachment styles influence marital dynamics under the strain of cancer. The implications for clinical interventions emphasize the importance of fostering secure attachment behaviors in couples therapy, particularly in contexts involving significant health challenges.

**Disclosure of Interest:**

None Declared

